# Why Does *Piscirickettsia salmonis* Break the Immunological Paradigm in Farmed Salmon? Biological Context to Understand the Relative Control of Piscirickettsiosis

**DOI:** 10.3389/fimmu.2022.856896

**Published:** 2022-03-21

**Authors:** Marco Rozas-Serri

**Affiliations:** Pathovet Labs, Puerto Montt, Chile

**Keywords:** piscirickettsiosis, *Piscirickettsia salmonis*, immunology, vaccines, control, SRS, salmonids

## Abstract

Piscirickettsiosis (SRS) has been the most important infectious disease in Chilean salmon farming since the 1980s. It was one of the first to be described, and to date, it continues to be the main infectious cause of mortality. How can we better understand the epidemiological situation of SRS? The catch-all answer is that the Chilean salmon farming industry must fight year after year against a multifactorial disease, and apparently only the environment in Chile seems to favor the presence and persistence of *Piscirickettsia salmonis*. This is a fastidious, facultative intracellular bacterium that replicates in the host’s own immune cells and antigen-presenting cells and evades the adaptive cell-mediated immune response, which is why the existing vaccines are not effective in controlling it. Therefore, the Chilean salmon farming industry uses a lot of antibiotics—to control SRS—because otherwise, fish health and welfare would be significantly impaired, and a significantly higher volume of biomass would be lost per year. How can the ever-present risk of negative consequences of antibiotic use in salmon farming be balanced with the productive and economic viability of an animal production industry, as well as with the care of the aquatic environment and public health and with the sustainability of the industry? The answer that is easy, but no less true, is that we must know the enemy and how it interacts with its host. Much knowledge has been generated using this line of inquiry, however it remains insufficient. Considering the state-of-the-art summarized in this review, it can be stated that, from the point of view of fish immunology and vaccinology, we are quite far from reaching an effective and long-term solution for the control of SRS. For this reason, the aim of this critical review is to comprehensively discuss the current knowledge on the interaction between the bacteria and the host to promote the generation of more and better measures for the prevention and control of SRS.

## Piscirickettsiosis: The Usual Enemy

Piscirickettsiosis (SRS) is caused by the Gram-negative facultative intracellular bacterium *Piscirickettsia salmonis*, which belongs to the subdivision of gamma-Proteobacteria with *Coxiella* and *Francisella* genera (1). *P. salmonis* has been confirmed as the causative agent for SRS in coho salmon (*Oncorhynchus kisutch*), Atlantic salmon (*Salmo salar*), and rainbow trout (*Oncorhynchus mykiss*) in Norway, Canada, Scotland, Ireland, and Chile (1). However, SRS especially affects salmon farming in Chile. In 2020, SRS was responsible for 47.8% and 67.3% of mortality related to infectious diseases and 11.7% and 10.7% of total mortality in the production of Atlantic salmon and rainbow trout, respectively (2). The most important determinants of the prevalence of SRS are the number of infected farms in upstream waters, followed by seawater salinity and temperature (3). On downstream farms of infected farms, the prevalence of SRS at 25 weeks of the cycle was close to 100%; while on farms with little or no exposure to infected upstream- farms, the prevalence only reached ~10% at week 56. Similarly, the prevalence of SRS within a group of concessions or “neighbourhoods” reached 100% at an average of 46 weeks after fallowing (4). 

At the same time, we observed an average reduction of 18.3% in the volume of antimicrobials used in the Chilean salmon industry between 2016 and 2019, although during 2020, there was a slight increase of 7.6% compared to 2019 (5). This is very important because a median of 93.5% of the total volume of antimicrobials used between 2017 and 2020 was used for SRS control. These results could be a consequence of the positive impact of the health strategy for SRS control based on a specific official epidemiological surveillance program, improved genetic resistance plans, regulatory changes, and good production practices, including animal welfare aspects, lower stocking densities, fallow periods, vaccination, among other practices. However, the slight increase observed in 2020 is an early warning indicator that has been observed, along with the detection of *P. salmonis*, the presentation of earlier clinical findings of SRS after transfer of smolts to seawater, and increased SRS-related mortality, all of which are indicators that support the relative efficacy of commercially available vaccines to control the disease.

**Figure 1:**
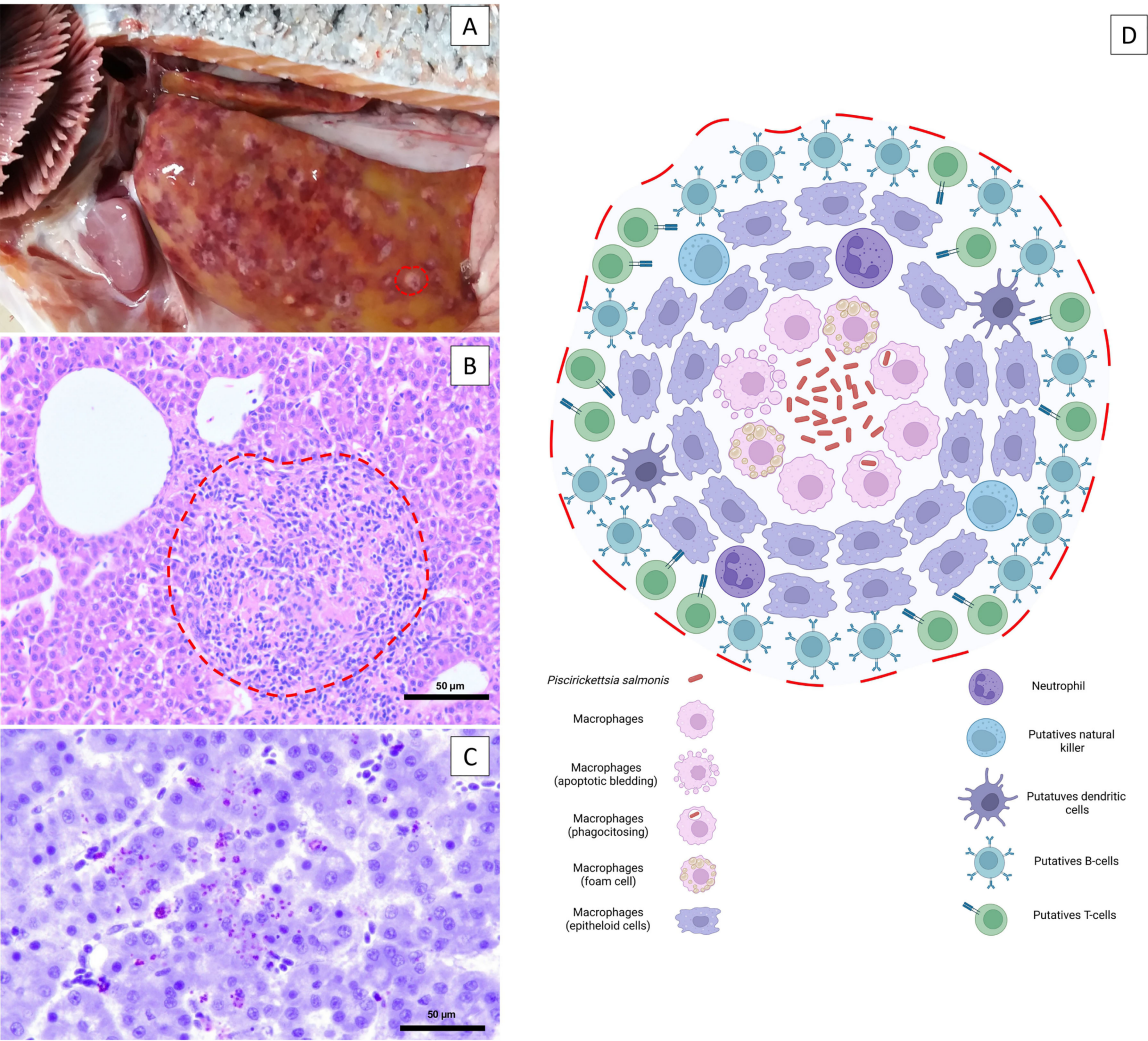
Macroscopic pathology and microscopic lesions in the liver associated with SRS infection. **(A)** Pale liver with subcapsular and circular reddish and gray–yellow mottled areas approximately 1-6 mm in diameter. The dashed red line delineates the borders of a gray–white discrete nodule characteristic of *P. salmonis* infection. **(B)** Multifocal necrosis of hepatocytes, diffuse infiltration of inflammatory cells and focal granuloma and/or multifocal coalescent granulomas. The dashed red line delineates the borders of a focal granuloma. Bar. 50 µm. **(C)** Immunolocalization of extracellular *P. salmonis* in the central zone of necrotic lesions and intracellular *P. salmonis* within macrophages using immunohistochemistry. Bar. 50 µm. **(D)** The yellowish-white nodules are granulomas typically consisting of a central necrosis with the presence of the bacteria, surrounded by macrophages at different stages, neutrophils, putative dendritic cells, and natural killer cells; all of these are surrounded by lymphocytes (putative T- and B-cells).

*P. salmonis* enters fish mainly through mucosal surfaces such as the skin and gills and, to a lesser extent, through the intestine (6–8). *P. salmonis* can even cross the intact skin in healthy fish (7), and although *P. salmonis* is not significantly negatively affected under exposure to the skin mucus of rainbow trout and Atlantic salmon over time, the cytotoxic effect of the bacterium pre-exposed to salmonid skin mucus is delayed (9). Rozas-Serri et al. (8) first reported a cohabitation challenge for *P. salmonis*, which was used to comparatively describe SRS pathogenesis using LF-89 and EM-90 isolates. This study concluded that the pathogenesis of postsmolt Atlantic salmon infected with LF-89-like and EM-90-like *P. salmonis* isolates was different. Fish infected with EM-90 showed higher cumulative mortality and a shorter time to mortality than fish infected with LF-89. EM-90 isolate produces an acute systemic and hemorrhagic disease characterized by lesions in all internal organs, whereas the most frequent internal lesions in fish infected with LF-89 show the more classical pathological picture of SRS with splenomegaly, renomegaly, large yellowish-white nodules in the kidney and liver, fibrinous pseudomembranes in the liver and heart, hydropericardium and ecchymosis in the liver (**Figure 1**). By histology, the yellowish-white nodules were found to be granulomas typically consisting of central necrosis along with the presence of bacteria and were surrounded by macrophages at different stages, neutrophils, putative dendritic cells (DCs), and natural killer cells; finally, all nodules are surrounded by putative T- and B-cells (**Figure 1**). However, a detailed molecular and cellular characterization of the granuloma arising during acute and chronic *P. salmonis* infection remains incomplete, mainly because antibodies to identify the different cell populations with confidence do not exist or are not accessible. *P. salmonis* induces flagellin-dependent tlr5 activation, which results in the up-regulation of *tnfa, il1b, il8* and *il16* (10–13), and promotes an intense proinflammatory response supported by leukocyte activation, and chemotaxis of neutrophils and macrophages towards the site of infection. At the same time, fish infected with *P. salmonis* showed up-regulation of *relb* and down-regulation of *nfkbiz*, which determine the activation of the NF- kB pathway (10, 12), promoting the up-regulation of genes encoding acute phase proteins (*lect2, ltbr4, gpr84* and *gpr43*) and chemokines (*cxcl7, cxcl9, cxcl10, cxcl12* and *cxcr1*) (10, 12, 13). Fish from both groups showed alterations in different blood biochemical parameters related to liver and kidney function, although the most severe alterations were present in fish infected with EM-90. In addition, the presence of *P. salmonis* was detected in the gills of coinhabiting fish of both groups at 21 days post-infection, confirming that the gills are the main point of entry of the bacterium into the host. Furthermore, the detection of the bacterium in the gills at 21 dpi and the occurrence of the first deaths at 36 dpi in fish with EM-90 and at 40 dpi in fish with LF-89 could indicate that these isolates have incubation periods of 15 and 20 days, respectively.

Cohabitation and immersion challenges are generally considered a better strategy compared to intraperitoneal injection challenges in terms of mimicking natural infection (8, 14, 15). There are only small differences between the intraperitoneal and cohabitation challenge models considering mortality and pathological changes (8, 14, 15). Meza et al. (14) reported that the incubation period was followed by an acute outbreak, with mortality reaching 100% in both challenged groups. Conversely, Rozas-Serri et al. (8) described a cumulative mortality of 84% and 100% in fish infected intraperitoneally with LF-89 and EM-90, respectively, but in fish infected using a cohabitation challenge model, the cumulative mortality reached 70 and 95%. In both studies, the pathological and histopathological changes were more visible during the clinical phase of the disease. Nevertheless, the death of fish in the cohabitant group was recorded earlier in the study from Meza et al. (14) than Rozas-Serri et al. (8) (28 dpc vs. 36 dpc, respectively), which could be due to the environmental variables of the experimental design; e.g., fish were kept in seawater at 32‰ and 15°C in the first case, while in the second case, the study was conducted in brackish water at 15‰ and 12°C. Finally, Long et al. (15) demonstrated that in tanks containing infected Atlantic salmon, using the immersion challenge model, the bacterium was first detected at 12 dpi, and levels peaked at 36 dpi, 3 days before the mean day of death. From 44 dpi onward, *P. salmonis* levels in the water column remained close to the detection limit and coincided with the cessation of acute mortality. Thus, the highest levels of *P. salmonis* are shed shortly before death, and Atlantic salmon experiencing an SRS outbreak are most infectious between 18 and 42 dpi.

Commercial vaccines are dominated by bacterin, recombinant (1) and attenuated live bacterial vaccines, administered primarily in water-in-oil emulsions (25) **(** **Table 1** **)**. Regardless of the species of salmon farmed, these vaccines against SRS have not been fully protective in reducing total mortality or in delaying the time to onset of the first SRS outbreak under field conditions in Chile (1, 26, 27). Oral immunization is an attractive alternative, primarily because of its lower cost and ease of administration to farmed fish. It has been suggested that several oral immunizations for SRS are essential to maintain the humoral adaptive immune response in farmed fish, expressed in terms of SRS-specific antibodies, and to keep the fish protected throughout the entire production cycle (22, 28). This scenario might be biologically plausible in the case of diseases caused by viruses or extracellular bacteria, but the facultative intracellular nature of *P. salmonis* requires that a truly effective vaccine must activate the cell-mediated adaptive (CMI) response for a sufficiently long time to protect the salmon population throughout the production cycle in seawater. Unfortunately, *P. salmonis* precisely modulates and compromises the CMI response in infected fish as a mechanism to escape the host defense (10–13, 29, 30). Other intracellular bacteria of importance in aquaculture, such as *Renibacterium salmoninarum* and *Edwardsiella tarda*, modulate CMI in a similar way. Rozas-Serri et al. (31) demonstrated that load of *R. salmoninarum* had a significant positive correlation with the down-regulation of *ifnγ, eomes, tbet, gata3, il2, il12, cd8* and *mpeg1* (perforin) in the head-kidney of Atlantic salmon pre-smolts; consequently, *R. salmoninarum* did not trigger a CMI response in fish infected at 11 or 15°C. Yamasaki et al. (32) showed the important role of CMI rather than humoral immunity against *E. tarda* infection in ginbuna cross carp. Bacterial clearance in the kidney and spleen was observed after elevated cytotoxic activity and increased numbers of CTLs, but *E. tarda*-specific antibody titers did not increase until after bacterial clearance, suggesting that induction of humoral immunity is late to provide protection. The length of time that CD8+ cells remain active to “kill” infected cells following infection with *P. salmonis* and/or vaccination is unknown, so optimal levels of CD8+ cell activation capable of conferring protection in salmon are also unknown. Moreover, the extent to which mucosal vaccination can evoke protective immunity against intracellular replicating bacteria remains unclear, unlike in the case of viral and extracellular bacterial infections, in which protective mechanisms have been widely studied (33, 34). Hence, the practical conditions under which intracellular vaccination translates into protective CMI responses have not been determined for SRS vaccines.

**Table 1:** Summary of experimental studies on efficacy evaluation of injectable and oral vaccines, types of antigens used and availability of commercial vaccines.

Vaccines	Antigen/laboratory	Vaccine type	Valence	Vaccine efficacy	Reference
Experimental	Bacterin	Inactivated whole-cell	1	Inconsistent results	Smith et al., (16)
	Outer surface protein A	Recombinant subunit	1	83% RPS	Kuzyk et al., (17)
	Whole genome	DNA	1	Mortality 80%	Miquel et al., (18)
	Bacterin	Inactivated whole-cell (heat)	1	70,7% RPS	Birkbeck et al., (19)
		Inactivated whole-cell (formalin)	1	49,6% RPS	
	Hsp60/70	Recombinant subunit	1	Mortality 8%	Wilhem et al., (20)
	Hsp60/70 + FlgG	Recombinant subunit	1	94,5% RPS	Wilhem et al., (21)
	TbpB MltB	Recombinant subunit	1	85% RPS	
	Omp27 FlaA	Recombinant subunit	1	10,4% RPS	
	Inactivated whole-cell, P. salmonis strain PS2C	Bacterin formulated in micromatrix for oral delivery	1	Protection by 1800 degree days	Tobar et al., (22)
	P. salmonis LF-89 bacterial membranes	bacterial proteoliposome + Montanide ISA 760 VG adjuvant water-in-polymer	1	46,1% RPS; 36,3% ARR; NNT = 3	Caruffo et al., (23)
	P. salmonis LF-89 bacterial membranes	bacterial proteoliposome + Montanide ISA 763 AVG adjuvant, water-in-oil	1	20,7% RPS; 16,3% ARR; NNT = 7	
	Immunogenic protein fraction 1	P1 - Immunogenic protein fractions + Montanide ISA 763 AVG adjuvant 1:1 oil-in-PBS	1	89,6% RPS	Pontigo et al., (24)
	Immunogenic protein fraction 2	P2 - Immunogenic protein fractions + Montanide ISA 763 AVG adjuvant 1:1 oil-in-PBS	1	8,3% RPS	
	Immunogenic protein fraction 3	P3 - Immunogenic protein fractions + Montanide ISA 763 AVG adjuvant 1:1 oil-in-PBS	1	11,5% RPS	
Commercial	Agrovet Ltda.	Inactivated whole-cell	1	N.I	S.A.G. 2021
	Agrovet Ltda.	Inactivated whole-cell	3	N.I	
	Agrovet Ltda.	Inactivated whole-cell	4	N.I	
	Agrovet Ltda.	Inactivated whole-cell	5	N.I	
	Veterquimica S.A.	Inactivated whole-cell	1	N.I	
	Veterquimica S.A.	Inactivated whole-cell	2	N.I	
	Tecnovax Chile S.A.	Inactivated whole-cell	1	N.I	
	Tecnovax Chile S.A.	Inactivated whole-cell	2	N.I	
	Tecnovax Chile S.A.	Inactivated whole-cell	3	N.I	
	Tecnovax Chile S.A.	Inactivated whole-cell	4	N.I	
	Tecnovax Chile S.A.	Inactivated whole-cell	5	N.I	
	Elanco	Inactivated whole-cell	2	N.I	
	Pharmaq	Inactivated whole-cell	2	N.I	
	Pharmaq	Inactivated whole-cell	3	N.I	
	Pharmaq	Inactivated whole-cell	4	N.I	
	Pharmaq	Live-attenuated	1	N.I	
	Centrovet Ltda	Inactivated whole-cell	1	N.I	
	Centrovet Ltda	Inactivated whole-cell	2	N.I	
	Centrovet Ltda	Inactivated whole-cell	3	N.I	
	Centrovet Ltda	Inactivated whole-cell	4	N.I	
	Centrovet Ltda	Inactivated whole-cell	5	N.I	
	Intervet Chile Ltda.	Recombinant subunit	2	N.I	
	Intervet Chile Ltda.	Recombinant subunit	3	N.I	
	Intervet Chile Ltda.	Recombinant subunit	4	N.I	
	FAV	Inactivated whole-cell	2	N.I	
	FAV	Inactivated whole-cell	4	N.I	
	FAV	Inactivated whole-cell	5	N.I	

Commercial vaccine information was taken from the official records of the Agriculture and Livestock Service (SAG), Chile. There are no efficacy results under field conditions in farmed salmonids in Chile for any of these injectable vaccines, only the field results that the producing companies manage at the end of each culture cycle. RPS, relative percentage survival; ARR, absolute risk reduction; NNT, treatment necessary number.

Yamasaki et al. (35) also showed the importance of CMI against *E. tarda* infection using vaccine trials comparing the effects of live vs. formalin-killed bacteria. Live cell-vaccinated fish showed high survival rates, high IFN-γ and T-bet gene expression levels, and increased CTLs. On contrary, all bacterin-vaccinated fish died following *E. tarda* infection and induced high IL4/13a and IL-10 expression levels, whereas Th1-like responses were suppressed. Rozas-Serri et al. (29) showed that a bacterin *P. salmonis* vaccinated-fish exhibited MHCI, MHCII, and CD4 overexpression but a significant downregulation of CD8b and IgM, suggesting that the formalin-killed bacteria promoted the CD4^+^ T-cell response but did not induce an immune response mediated by CD8^+^ T cells or a humoral response. The level of SRS-specific antibodies generated by vaccines and/or parenteral or oral boosters does not correlate with the level of protection or a reduction in the SRS-related mortality rate under field conditions. For this reason, no significant differences are observed between fish receiving a pentavalent injectable vaccination plus an oral booster and those receiving the same regimen but without the oral booster (27). Similarly, there is also no difference in overall mortality or time to first outbreak in fish receiving one or more saltwater booster vaccines and fish not receiving the booster (26).

In the last four years, our research group has evaluated the immune response of fish under field conditions against different vaccines, including the live attenuated *P. salmonis* vaccine. Overall, the activation of the innate immune response and the cell-mediated adaptive response modulated by overexpression of *ifnγ, il2, il10, il12, il12, mhc1, cd4* and *cd8* in the head kidney of Atlantic salmon smolts at 24 hours postvaccination and 150-, 300-, 460- and 600-degree days before transfer to seawater were observed. These findings are frequently noted in fish farmed in open-flow water hatcheries at different water temperatures (between 7 and 14°C) and in recirculation aquaculture systems (RAS), but the response is always significantly better in fish kept at higher temperatures (> 12°C). Acute and chronic exposure to suboptimal temperatures often has suppressive effects on the immune response of fish, especially on adaptive immunity (36). Sanhueza et al. (37) demonstrated that behavioural fever in ectotherms leads to a neuroimmune interaction that could modulate the systemic inflammatory response during pathogen infection. Results from the last time point of the monitoring plan conducted at 1,800-degree days postvaccination (1,200-degree days in seawater) showed a reduction in *cd4* and *cd8* gene expression, a time that usually coincides with the onset of the first SRS outbreaks in Atlantic salmon seawater farms. The downregulation of *cd4* and *cd8* at this point would indicate a reduced CMI response, probably associated with the gradual use of the different immune components previously induced by the vaccine and/or associated with a natural challenge.

These results are consistent with the transient stimulation of the CMI response described by Vargas et al. (38) in fish immunized with the same vaccine under field conditions, as they observed increased gene expression of *ifna*, *ifnγ*, *cd4*, cd8a, *il10* and *tgfb* at 5 days postvaccination but a decreased response at 15 and 45 days postvaccination. However, the results of the latter study were obtained from fish vaccinated with an injectable booster vaccine in seawater when they were on average between 1 and 1.5 kg in weight. In Chile, 100% of Atlantic salmon smolts entering the sea are vaccinated with a vaccine containing the *P. salmonis* component, so these results could be influenced by pentavalent injectable primo-vaccination and natural challenge after entering seawater. Although we know that current vaccines do not sufficiently activate the CMI response to protect fish throughout the production cycle, current vaccines are important in the relative control of SRS. Nevertheless, a decrease in protection in terms of the time to the first detection of *P. salmonis* and SRS outbreaks, an increased number of therapies, and increased associated mortality, among other indicators, has been noted.

## *Piscirickettsia salmonis*: A Fastidious Bacterium

*P. salmonis* is a facultative intracellular bacterium because although it infects lymphoid cells such as macrophages, monocytes and nonlymphoid cells *in vitro* and *in vivo* (39, 40), it can be cultured *in vitro* in solid and liquid media enriched with cysteine (41). Cortés et al. (42) identified the metabolic characteristics of *P. salmonis* that differentiate the two main clades of the species and demonstrated that *P. salmonis* could benefit from different experimentally tested carbon sources in newly defined media. The genome of *P. salmonis* contains one circular chromosome of 3,184,851 bp and three plasmids, pPSLF89-1 (180,124 bp), pPSLF89-2 (33,516 bp) and pPSLF89-3 (51,573 bp) (43). The in silico analysis of several strains of *P. salmonis* showed an open pangenome of 3463 genes and a coregenome of 1732 genes (44). Plasmids are extrachromosomal circular or linear double-stranded DNA molecules that vary in size, and some bacteria contain plasmids with no obvious functions that are classified as cryptic. Ortíz-Severin et al. (45) identified four large cryptic plasmids in the *P. salmonis* reference strain LF-89, in addition to twelve putative virulence factors and two global transcriptional regulators. These plasmids would be predicted to be critical in the bacterium’s ability to adapt to the environment, as they encode proteins related to nutrient mobilization, transport, and utilization.

Mauel et al. (46) showed that *P. salmonis* isolated in different parts of the world, including the reference strain LF-89, are closely related to each other (over >99% similarity in their 16S, ITS and 23S genes), but the Chilean isolate EM-90 is unique and genetically divergent from the others. Later, these findings were confirmed with more evidence from evaluations of their genomic structures and phylogenetic relationships to support the existence of two genogroups of *P. salmonis*, LF-89 and EM-90 (44, 47–52). *P. salmonis* pangenome LF-89 and EM-90 show 148 and 273 unique proteins, respectively (44), and different geographical distribution patterns and susceptibilities of salmon species have been described (53). This notion has also been experimentally supported by studies based on multilocus sequence typing (MLST) and PCR amplification of 16S rRNA genes followed by restriction fragment length polymorphism (PCR-RFLP) typing (51, 54). Although different real-time TaqMan^®^ PCR assays have been used for several years by private diagnostic laboratories to identify and discriminate between LF-89 and EM-90 isolates in the Chilean salmon industry, a multiplex PCR protocol to differentiate both genogroups has recently been reported (52). *P. salmonis* can survive ≥120 h and replicate in cell cultures enriched with Atlantic salmon macrophages (55). This strategy would be induced by a limited lysosomal response that could be associated with *P. salmonis* host immune evasion mechanisms. Later, Pérez-Stuardo et al. (56) showed that IgM bead treatment promotes lysosomal activity in Atlantic salmon macrophage-enriched cell cultures infected with *P. salmonis* by reducing the lysosomal pH and increasing the proteolytic activity within the lysosome, reducing the bacterial load and the cytotoxicity induced by *P. salmonis*. Artificially induced iron deprivation during *P. salmonis* infection *in vitro* and *in vivo* using iron chelators generates a protective response of infected cells coincident with a reduction in bacterial load and cell damage (57, 58).

Although numerous virulence-related genes have been identified in *P. salmonis*, only a few encoded virulence factors have been characterized **(** **Table 2** **).** Proteolytic enzymes are important virulence factors because they are involved in cell invasion and intracellular proliferation. *P. salmonis* can synthesize and secrete siderophores (81), confirming its ability to utilize different sources of iron. In addition, *P. salmonis* has protease activity that increases significantly when the bacterium infects cells (62). At the same time, genomic islands have been described in the genomes of different *P. salmonis* strains (73), which could explain the different virulence levels of *P. salmonis* genogroups, pathogenesis (8, 14) and immune response observed in both fish experimentally challenged with different *P. salmonis* genogroups and vaccinated fish (11, 12, 14, 29). Intracellular bacterial pathogens use different strategies that allow them to adhere to, invade, and replicate in host cells and modulate intracellular processes such as membrane trafficking, signaling pathways, metabolism, and cell death and survival (85). One of the strategies used by these bacteria is secretion systems (SSs), which are complex multiprotein transmembrane nanomachines that form a channel that allows for the exportation of different molecules, including virulence effectors (86). *P. salmonis* is deficient in organelle trafficking/intracellular multiplication (Dot/Icm) secretion system genes and is classified as a type IVB secretion system (T4SS) (74, 75), which has been described as the main virulence mechanism of *Legionella pneumophila* and *Coxiella burnetii* and is responsible for their intracellular survival and replication (87). Temporal acidification of cell-free media results in overexpression of *P. salmonis* genes that inhibit phagosome-lysozyme fusion to prevent phagolysosome killing (74). Complementarily, the Sec-dependent pathway and Type 4B secretion system are biologically active during *in vitro P. salmonis* infection (76). The type VI (T6SS) and type III (T3SS) secretion system is also present in the *P. salmonis* genome, but no studies have demonstrated its functional mechanism.

**Table 2:** Virulence-related factors described in *P. salmonis*.

Virulence factor	Function	Reference
Lipopolysaccharide (LPS)	Endotoxicity	Vadovic (59); Fodorova (60); Vinogradov (61)
Proteases (metalloproteases, elastases, etc)	Protease activity. Bacterial cell invasion and intracellular proliferation	Figueroa (62)
Heat shock proteins	Molecular chaperone. Survival and replication within macrophages	Isla (63); Oliver (64); Oliver (65)
ISPsa2	Plasticity and adaptability	Marshall (66); Gómez (67)
Biofilms	Survival and persistence under stress conditions	Marshall (68); Levipan (69); Oliver (70); Santibañez (65)
Secreted extracellular products	Cytotoxicity	Rojas (71); Smith (72)
Genomic island (tcf, dnsa and liso)	Cytotoxicity	Lagos (73)
Dot/Icm proteins	Interference with the endosomal maturation process to ensure intracellular bacterial survival	Gómez (74); Labra (75); Cortés (76)
Secretion systems	Intracellular survival and/or replication (T3SS, T4SS, T6SS)	Gómez (74); Bohle (77); Ortiz-Severin (78)
OMV	Bacterial pathogenesis	Oliver (64); Lagos (79); Tandberg (80);
Toxins	Bacterial toxins	Oliver (64)
Iron metabolism	Iron metabolism	Calquin (81); Ortiz-Severin (78)
	Sidrophores metabolism	Calquin (81)
Pilus	Secretion system	Sánchez (82); Ortiz-Severin (78)
Flagellar	Motion/T3SS	Ortiz-Severin (78)
Stringent response	Survival under nutrient starvation and other related stresses	Zuñiga (83); Zuñiga (84); Ortiz-Severin (78)

Putative plasmid-encoded toxins are secreted in *P. salmonis* extracellular vesicles (EVs) (64, 79, 80, 88), which suggests a possible role in bacterial virulence of this important mobile genetic element. Leiva et al. (89) identified 35 unique proteins in serum EVs from *P. salmonis*-infected fish, including proteasome subunits, granulins and major histocompatibility classes I (MHC-I) and II (MHC-II). Therefore, these results suggest that the release of EVs could be part of a mechanism in which host stimulatory molecules are released from infected cells to promote an immune response. Additionally, other mechanisms of pathogenicity have been described in *P. salmonis*, such as outer membrane vesicles (OMVs) containing different proteins related to critical survival functions, plasmid-encoded toxins (64, 79, 80, 88) and type IV pili, which are filamentous structures on the bacterial surface important for adherence to host cell surfaces (82) **(** **Table 3** **)**. Biofilms play an important role in bacterial pathogenicity, as their physical and spatial arrangement impedes access to antimicrobials and increases their resistance to phagocytosis. *P*. *salmonis* forms viable, stable, and fish skin-mucus tolerant biofilms on abiotic surfaces and under conditions of severe nutrient starvation (69). The cytotoxic response of the salmon head kidney cell line to *P*. *salmonis* showed interisolate differences because LF-89 isolate biofilms were sensitive to Atlantic salmon skin mucus during early formation, whereas EM-90 isolate biofilms were more tolerant. Complementarily, Santibañez et al. (90) showed that the planktonic isolate EM-90 and the sessile LF-89 would generate the highest levels of virulence *in vitro* by the modulation of the proinflammatory response (*il1b, il8, nfkb*, and *ikba*). NaCl and Fe significantly increase biofilm production. Oliver et al. (65) suggested that the presence of Hsp60 (GroEL) in *P. salmonis* OMVs would insinuate that they may be important in interacting with host proteins and/or modulating biofilm formation.

**Table 3:** Major virulence-related proteins described in membrane vesicles of *P. salmonis* strain type LF-89.

Proteins	Description	Function
Pertussis_S1 superfamily	Pertussis toxin, subunit 1.	Toxin
Enterotoxin A superfamily	Heat-labile enterotoxin alpha chain	Toxin
OM_channels superfamily	Porin superfamily	Porin
OmpA	C-terminal domain of outer-membrane protein OmpA	Bacterial adhesion, invasion, or intracellular survival as well as evasion of host defenses or stimulators of pro-inflammatory cytokine production
Porin F	Peptidoglycan binding domains similar to the C-terminal domain of outer-membrane protein OmpA	Porin
VirB9/CagX/TrbG superfamily	VirB9/CagX/TrbG, a component of the type IV secretion system	A component of the type IV secretion system
CsrA superfamily	RNA-binding protein and a global regulator of carbohydrate metabolism genes	This protein is a RNA-binding protein and a global regulator of carbohydrate metabolizm genes facilitating mRNA decay
SrfB superfamily	This family includes homologues of SsrAB is a two-component regulatory system encoded within the Salmonella pathogenicity island SPI-2	This family includes homologues of SsrAB is a two-component regulatory system
FliH superfamily	Flagellar assembly protein FliH.	A component of flagella

*Legionella pneumophila* is an aquatic organism that interacts with amoebae and ciliated protozoa as natural hosts, and this interaction plays a central role in bacterial ecology and infectivity (70). Declerck et al. (91) demonstrated that while *L. pneumophila* was present in 100% of floating biofilms in anthropogenic aquatic systems, *Naegleria* spp. and *Acanthamoeba* spp. were present in 50-92% and 67-72% of floating biofilm samples, respectively. Recently, Labra et al. (92) demonstrated that *P. salmonis* is detected only in adults of the crustacean ectoparasite *Caligus rogercresseyi* and that it is present only transiently after removal of *P. salmonis*-infected hosts, but the bacterium is not detected in the chalimus stages or in planktonic larvae of the parasites. However, the relationship of *P. salmonis* with *Neoparamoeba perurans*, a cosmopolitan marine amoeba and causative agent of AGD, remains unclear.

## The Intracellular Invasion and Survival Strategy of *P. salmonis*

More information is now available to understand the process of invasion and survival of *P. salmonis* at the intracellular level (78, 83, 84). Although this knowledge remains to be validated *in vivo* using fish ideally challenged by cohabitation or immersion and under field conditions, it is an important step forward in the systematization and understanding of bacterial biology in host cells. However, like other intracellular bacteria in mammals and fish, we usually see that this battle between *P. salmonis* and fish defenses is won by the bacteria, probably because it has a wide arsenal of highly efficient virulence factors that allow it to evade the adaptive cell-mediated immune responses and modify the cell-autonomous immunity of the fish cells to its benefit, promoting a favorable environment for its replication and chronic maintenance in animals (8).

**Figure 2:**
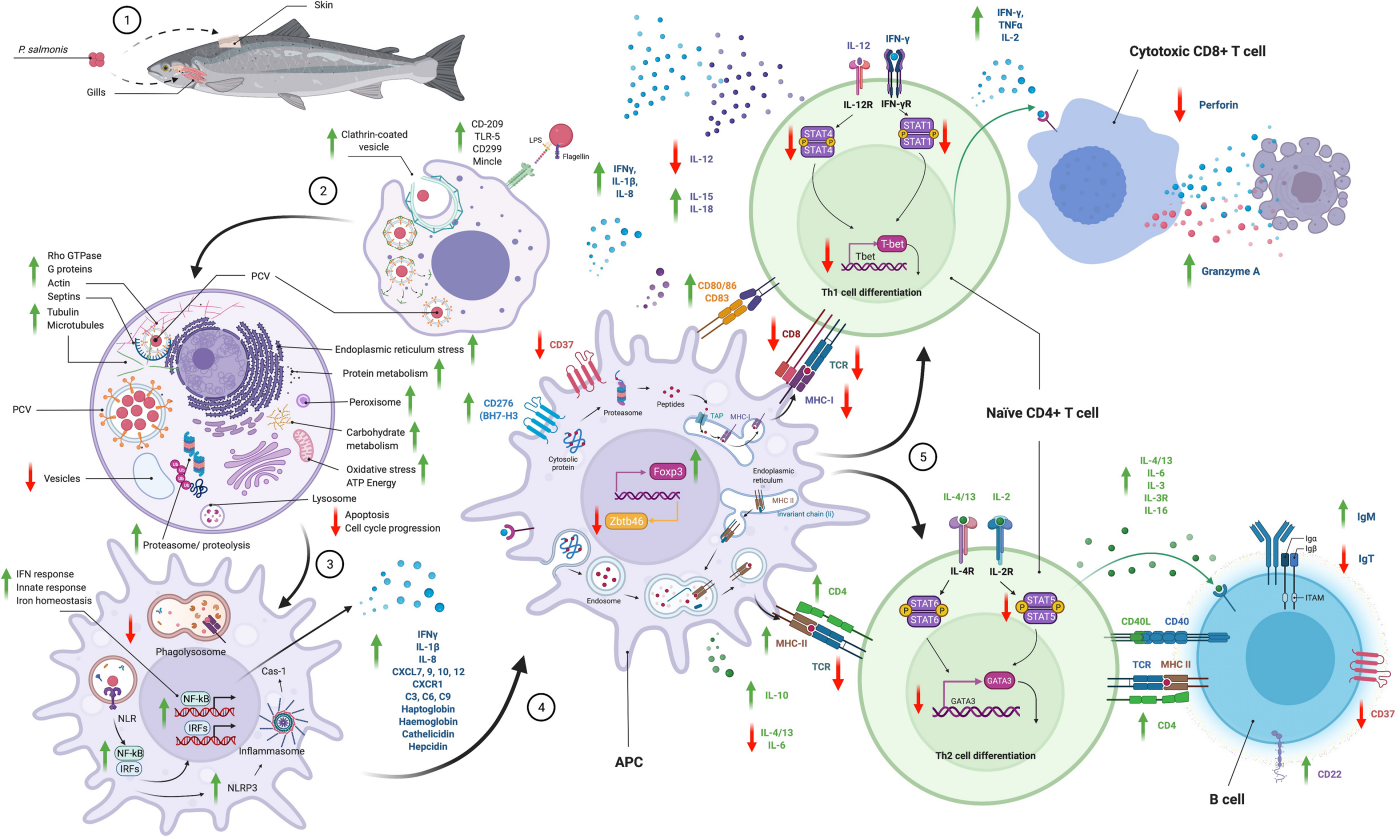
Schematic summary of the pathways used by *P. salmonis* to enter to the cell and the immune responses modulated. This information was consolidated with results descrbed in different *in vitro* and *in vivo* transcriptomic and proteomic studies. Phagocytosis, key in the life cycle of *P. salmonis*, is also its primary mode of pathogenesis. The interaction of *P. salmonis* with host cells has been described by different transcriptomic and proteomic studies *in vitro* and *in vivo* (10–13, 29, 30, 78, 89, 93–107). *P. salmonis* infection consists of an early or vacuolization stage and a late or spreading stage. (1) Several studies have reported that the main route of entry of the bacterium into the fish is through the gills and skin and, to a much lesser extent, the oral route. (2) *P. salmonis* is internalized by clathrin-dependent endocytosis into phagocytic cells. Pilus proteins, beta-hemolysin, and the T6SS are a characteristic finding of the vacuolization stage. The transport of carbohydrates, amino acids, peptides, iron, and other nutrients is increased inside the PCVs. (3) Once inside, a major reorganization of the cytoskeleton occurs by altering actin filaments, tubulins, myosins, and septins, and *P. salmonis* simultaneously promotes an inactive GTPase state. *P. salmonis* induces a significant inhibition of the antioxidant response that would promote the generation of an oxidative niche. Overall, *P. salmonis* alters cytoskeletal remodeling, intracellular transport, organelle organization, vesicle and endosome trafficking and early endosomal components. The antigen recognition (LPS, flagellin, etc.) is conducted by macrophage and dendritic cell (DC) PRRs, such as TLR5, DC-SIGN or CD209, C-type lectin (CD299, Mincle), and NLR. DCs play an important role in the response to *P. salmonis* by modulating NF-kB activation, pathogen recognition, phagocytosis and the production of cytokines and chemokines led by the IFN-mediated response that promotes Th1 polarization of T cells. At the same time, to enhance its survival in infected cells, *P. salmonis* upregulates IL-10 but downregulates IL-12, which promotes Th1 polarization. *P. salmonis* induces flagellin-dependent activation of TLR5, resulting in TNFα, IL-1β and IL-8 production. *P. salmonis* promotes the expression of antimicrobial peptides, such as hepcidin and cathelicidin, and acute-phase components such as haptoglobin, hemoglobin, collectrins, mannose-bound protein C, complement components (C3, C6, C9) and CD163. All these findings confirm that *P. salmonis* creates a specific environment that promotes their survival and replication in macrophages. During the propagation stage, the size and number of *P. salmonis* cells and vesicles increase, and nutrient availability is restricted, initiating a stringent response. Biosynthetic processes are increased in both *P. salmonis* and host cells, as are iron acquisition, iron transporter proteins and acute-phase responses. Expression of Dot/Icm T4SS genes, toxins, effector proteins, mobilome, transposons, and phage-related proteins is increased, probably in preparation for exiting the cell. Many virulence factors upregulated in both infection stages correspond to plasmid-encoded proteins, which supports the hypothesis of the importance of *P. salmonis* plasmids in the infective process. *P. salmonis* modulates the immune response to intracellular pathogens by promoting cell cycle proliferation and suppressing apoptosis and by altering vesicle trafficking and paracellular permeability. *P. salmonis* alters peroxisome activity as part of its infection strategy, thereby inducing an altered cellular redox balance, inflammation, and immune response. Moreover, *P. salmonis* reduces the rate of protein degradation by the ubiquitin proteasome system related to the response to cellular and endoplasmic reticulum stress-associated unfolded proteins as a mechanism to increase its survival within host cells. (4) Processing and presentation of antigens. T cells recognize only antigen fragments that are bound to MHC-I or MHC-II on APCs. Antigens presented by MHC-I are processed through the proteasome and transferred to the endoplasmic reticulum by a transporter associated with antigen processing (TAP) where they associate with MHC-I and are finally transported to the cell membrane. MHC-II-presenting antigens are incorporated into cells by endocytosis, digested in lysosomes and loaded onto MHC-II molecules prior to migration to the cell surface. However, *P. salmonis* inhibits the MHC-I pathway (*mhc1, cd8, tcra, tcrb* gene underexpression) but activates the MHC-II pathway (*mhc2, cd4*), so its strategy is to evade the CD8+ T cell-mediated immune response. *P. salmonis* increases the expression of important co-stimulatory molecules on the macrophage surfaces (*cd80/86, cd83*) and reduces the expression of *zbtb46*, a transcriptional factor that inhibits APCs maturation (5) T cell activation and differentiation. CD4+ T helper cells can differentiate into Th1, Th2, Th17 and Treg populations, which play different roles in the immune response. IL-12 promotes the differentiation of CD4 cells into Th1 cells to eliminate intracellular pathogens, while IL-2, IL-4, IL-13A, and IL-10 promote the differentiation of Th2 cells in response to bacteria and extracellular parasites. However, *P. salmonis* promotes IFN-γ production but reduces IL-12 production, reduces the expression of Th1 polarization-specific transcription factors (*tbet*, *eomes*) and, while promoting granzyme A expression (*gzma*), reduces perforin (*mpeg1*) production. In addition, *P. salmonis* increases the expression of Treg polarization-specific transcription factor (*foxp3*) suggesting the activation of an immune tolerance response.

*P. salmonis* can infect, survive, and replicate primarily within the cytoplasmic vacuoles of macrophages and polymorphonuclear leukocytes without inducing a characteristic cytopathic effect (40) (**Figure 2**), although its replication in other dedicated phagocytic cells, such as DCs and B cells, remains to be elucidated. While macrophage biology has been characterized in mammals, macrophage differentiation and activation in teleost remain to be adequately described. Macrophages have a high degree of plasticity and can be activated by the classical (M1 or pro-inflammatory) or alternative (M2 or anti-inflammatory) pathway (108). M1 macrophages are activated by IFN-γ and TNF-α and produce proinflammatory cytokines and ROS to protect against pathogens (109). M2 macrophages are activated by *il4/il13, il10, tgfb* and are characterized by inducing lower microbicidal activity, immunosuppression and promoting cell growth and wound healing (110, 111). In teleost monocytes/macrophages, inducible nitric oxide synthase (iNOS) is an M1-type marker and arginase 2 is an M2-type marker (112). Smith et al. (113) observed a change in the morphology, phagocytic ability, and miRNA profile of Atlantic salmon head-kidney leukocytes (HKLs) *in vitro*, showing that the cells differentiate from “monocyte-like” (Day 1) to “macrophage-like” (Day 5). At the same time, the abundance of some miRNAs in EVs was significantly different from the abundance of miRNAs in HKLs, suggesting that these miRNAs are involved in the immune response and/or macrophage activation (114). Smith et al. (115) revealed major changes in the transcriptome of HDLs at day 1 and 5, including changes in the expression of macrophage and immune-related transcripts (*csf1r, arg1, tnfa, mx2*), lipid metabolism (*fasn, dhcr7, fabp6*) and transcription factors related to macrophage function and differentiation (*klf2, klf9, irf7, irf8, stat1*). Thus, the HKLs population differentiates *in vitro* to become macrophages without the addition of exogenous factors.

Macrophages infected with *P. salmonis* induce an anti-inflammatory milieu, probably involved in the development of its bacterial virulence mechanism to ensure replication and survival (11, 12, 93). The impact of an infection depends on the balance between the ability of macrophages to recognize and destroy bacterial pathogens and the ability of pathogens to disrupt the functions of these macrophages (112). Intracellular pathogens share several mechanisms of subversion of host immune responses (116), such as: (a) evasion of host immune recognition such as modulation of microbial surfaces, secretion of immunomodulators, antigenic variation, and concealment in target cells or tissues; and (b) modulation and suppression of host immune responses such as evasion of phagocytosis, innate immune receptors, the complement system, cytokines or chemokines, inhibition of apoptosis, resistance to host effector mechanisms, and induction of inappropriate immune responses such as immunosuppression and induction of Tregs. One of the most important strategies used by *P. salmonis* is the evasion of phago-lysosomal degradation (55, 56, 74, 76), but it is also capable of activating a mechanism to subvert host defense by inhibiting fusion with the host lysosomal compartment and altering lysosomal pH (74). Moreover, *P. salmonis* can escapes into the cytosol and replicate in the host cell cytoplasm (39). *P. salmonis* can resist host effector mechanisms because it can persist in macrophages (63), inhibiting apoptosis (40), inhibiting oxidative stress processes, and promoting cell cycle (10, 12, 13, 78, 83). Finally, *P. salmonis* induces an inappropriate immune response, immunosuppression (10–13, 29), and differentiation of Tregs (94).

Upon entering macrophages through phagocytosis, *P. salmonis* orchestrates the formation of vacuoles called *P. salmonis*-containing vacuoles (PCVs), and it evades the lysosomal degradation pathway by escaping into the cytoplasm (39, 40, 74). Clathrin and the actin cytoskeleton play pivotal roles in *P. salmonis* internalization and multiplication, respectively (12, 95, 96). PCVs are generated because *P. salmonis* fully exploits disorganized and *de novo* synthesized actin in its favor, generating tridimensional vacuoles, apparently made exclusively of actin during the later stages of infection (95). Clathrin-mediated endocytosis is a well-documented portal of entry for many intracellular bacteria and viruses (117). Phagocytosis, the key to the life cycle of *L. pneumophila* and *P. salmonis*, is also involved in their pathogenesis. In both cases, a key part of the pathogenesis process are the genes and gene complexes that they use to secrete morphological structures (e.g., pili) or reactive proteins. Several proteins produced by *L. pneumophila* allow it to evade the cellular immune response and replicate inside macrophages. Some of these proteins are the virulence factor BipA/TypA and the heat shock protein ClpB, both of which have been extensively characterized (118). Isla et al. (63) showed a significant increase in the expression of ClpB and BipA proteins during infection of SHK-1 cells by *P. salmonis*, whereby these virulence factors would be used by the bacterium to evade cell degradation mechanisms and promote its replication inside macrophages.

Inside the cells, *P. salmonis* alternates between a replicative and a stationary phase in which a strict response is activated (78, 83, 84). *P. salmonis* induces an interruption of translation during its intracellular replication, and the Dot/Icm secretion system type IVB plays an important role during this process (83); however, there are still many unexplored genes that are active during intracellular infection of the bacterium. Global proteomic profiling to identify differentially expressed proteins in macrophage-like cells of Atlantic salmon challenged with *P. salmonis* at different stages of infection has been described (78, 84). These results confirm previous findings at the transcriptomic level, and they are valuable to understand the biological pathways of *P. salmonis* infection *in vitro* at the protein level, but the need to obtain this knowledge from experimental and naturally infected fish *in vivo* remains. A model for the infective process of *P. salmonis* based on a vacuolization stage and a propagation stage can be proposed from the transcriptomic and proteomic results (10, 12, 13, 30, 78, 83, 84, 93, 95–97) (**Figure 2**).

The stringent response is one such adaptive mechanism through which *P. salmonis* can survive under nutrient starvation and other related stresses (119). It has been suggested that amino acid and fatty acid starvation triggers RelA and SpoT to produce the alarmones guanosine tetra- and pentaphosphate ((p)ppGpp) in different bacteria (120). Zúñiga et al. (84) described the upregulation of these key genes during the *P. salmonis* stationary phase and intracellular growth, including genes encoding the two-component sensor kinase LetS, the stationary phase sigma factor RpoS, and the (p)ppGpp synthetase and/or hydrolase RelA and SpoT, which have all been shown to play an important role in regulating virulence in *L. pneumophila* and *C. burnetii* (121). During the late stages of infection, the LCV becomes disrupted, leading to bacterial egress into the cytosol. Upon nutrient depletion, RelA and SpoT are triggered, leading to increased levels of ppGpp, which triggers phenotypic transition into stationary phase (122).

## *Piscirickettsia salmonis* Evades the Cell-Mediated Adaptive Immune Response: Is It Checkmate?

Microbial infections are characterized by a constant interplay between pathogens and hosts, with pathogens exploiting various host functions during infection and hosts reacting with appropriate defense responses. Therefore, understanding host–pathogen interactions is crucial for the development of effective vaccines and therapies. Antigen-presenting cells (APCs) such as macrophages and dendritic cells (DC) sense bacteria through pathogen recognition receptors (PRRs) activated by pathogen-associated molecular patterns (PAMPs). Among the main PRRs and PAMPs are Toll-like receptors (TLRs) and flagellin, respectively (**Figure 2**). *P. salmonis* has a complete and organized set of flagellar genes, although no structural flagellum has ever been reported for this bacterium (123). Flagellin binding to TLR5 activates the MyD88-dependent pathway, leading to the activation of IRAK-1/4 and TRAF-6 and resulting in the activation of NF-κB, which induces the expression of proinflammatory cytokines (124). Enrichment analyses of the differentially expressed genes revealed several central signatures following infection, including positive regulation of TLR5 signaling, which converged at the NF-kB level to modulate the proinflammatory cytokine response (12). Flagellin from different bacteria induces the upregulation of cathelicidin (camp) *in vitro*, and TLR5 is involved in the signaling pathway (125). Rozas-Serri et al. (12) observed upregulation of tlr5 and camp in fish infected with *P. salmonis*. Lately, Muñoz-Flores et al. (126) showed that MyD88 is an essential adaptor protein in the activation of the *P. salmonis* flagellin-mediated TLR5M/TLR5S signaling pathway during *in vitro* infection. Several *in vitro* and *in vivo* assays have suggested a possible use for flagellin as an immunostimulant or vaccine adjuvant (127). The flagellar protein FlgG of *P. salmonis* achieved the highest level of protection, with a relative percent survival (RPS) of 95% (21). Recently, González-Stegmaier et al. (128) demonstrated that the full recombinant flagellin B from *Vibrio anguillarum* (rFLA) and its recombinant D1 domain (rND1) induced the expression of genes involved in an inflammatory response quickly and for a short time and that these effects can occur when the molecules are used alone or in combination with a commercial vaccine against *P. salmonis*.

*P. salmonis* induces significant cytoskeletal reorganization but decreases lysosomal protease activity and causes the degradation of proteins associated with cellular stress (12, 95) (**Figure 2**). Infection with *P. salmonis* also delays protein transport, antigen processing, vesicle trafficking and autophagy but promotes cell survival and proliferation and inhibits apoptosis (12). As *P. salmonis* has been shown to undergo both replication and degradation within rainbow trout head kidney macrophages, bacterial antigens could potentially be presented by the MHC-II system (39). Alternatively, as the bacterium has been shown to inhibit the fusion of phagosomes and lysosomes, *P. salmonis* could remain within phagosomes for replication followed by subsequent release or escape (74).

It has been reported that IFN would promote increased expression of APC-related markers (MHC-I and MHC-II) and down-regulation of ZBTB46 in rainbow trout, which is a transcriptional factor that inhibits APC maturation (129, 130). Recently, IFN-γ has been shown to promote the expression of cell surface markers (CD80/86, CD83 and MHC-II) and decrease the expression of zbtb46 in mononuclear splenocyte subpopulations of Atlantic salmon (98). Taken together, these results would confirm that IFN-γ promoted by *P. salmonis* modulates the interaction between APCs and T cell polarization, and that an optimal antigen presentation process is essential for the activation of a protective CMI response. Nevertheless, it is necessary to complement these findings with the expression of the counterpart molecular components of these markers on T cells, such as CD28 and CTLA4. Furthermore, an up-regulation of the transcription factor *foxp3* has been described in rainbow trout splenocytes co-cultured with both IFN-stimulated cells and cells stimulated with *P. salmonis* proteins (94), demonstrating an intercommunication between APCs and lymphocytes that would promote a polarization towards a Treg phenotype. However, different transcriptomics results have shown that *P. salmonis* effectively modulates the upregulation of MHC-II and CD4 T cells and antibody responses, but these indicators are not correlated with the protection of vaccines or better survival rates in field conditions. Moreover, *P. salmonis* induces a downregulation of the CMI response led by cytotoxic CD8 T cells.

Transcriptome analysis has revealed a global translation shutdown during intracellular growth of *P. salmonis*, and it has been proposed that intracellular *P. salmonis* alternates between a replicative phase and a stationary phase during which a stringent response is activated (83). *In vitro* and *in vivo* functional genomic studies of *P. salmonis* infection in salmon have focused on changes in coding gene expression (10, 12, 13, 30, 83, 97–99), small/microcoding RNA expression (100), and long noncoding RNA expression (96) **(** **Table 4** **)**. In addition, Leiva et al. (89) showed a significant DNA methylation alteration in *P. salmonis*-infected Coho salmon with a temporal pattern during infection. The number of differentially methylated regions and the associated metabolic pathways would support the hypothesis that epigenetic changes in the genome of infected Coho salmon could be modulated by *P. salmonis*. Trout skeletal muscle is an immunologically active organ that can implement an early immune response against *P. salmonis* (101). In fact, this response could be differentially regulated by cortisol, which could lead to bacterial outbreaks in muscle under stress conditions (102, 103). A common transcriptional response associated with clathrin-mediated endocytosis and iron homeostasis has been shown in different tissues (12, 96, 97, 104, 105). Dual global transcriptomic analysis revealed a bacterial dependency on host metabolism and nutrient accessibility (30). Interestingly, genome-wide comparisons of *P. salmonis* revealed the absence of the biosynthetic pathway for valine, leucine, and isoleucine. When these amino acids are restricted, bacterial growth dynamics are significantly impaired, but this condition is reversed when amino acids are supplemented again. All these transcriptomic analyses have provided evidence of host biological processes, cellular components, and molecular functions during *P. salmonis* infection to evade the immune response, induce significant cytoskeletal reorganization, and promote intracellular survival and replication. Nucleotide-binding oligomerization domain-like receptors, or NOD-like receptors (NLRs), are intracellular receptors responsible for recognizing pathogens. Pontigo et al. (106) described two different isoforms of SsNLRC3 in *P. salmonis*-infected Atlantic salmon, whereby the isoform might have a different function in the recognition of bacterial ligands during infection. However, these results suggest that, similar to *L. pneumophila* (131), *P. salmonis* promotes pyroptosis, a type of programmed cell death associated with intracellular pathogen infection characterized by inflammasome formation, caspase-1 activation and proinflammatory cytokine production.

**Table 4:** *In vivo* transcriptomic studies describing innate and adaptive immune responses in Atlantic salmon and Coho salmon infected with *P. salmonis*.

Challenge model	Specie	Size	Isolate/doses	Taget tissue	Expression	Analysis	Reference	Main discovery
I.P challenge: water temperature and salinity not reported, but it is assumed that it is freshwater because the authors speak about Atlantic salmon “parr”	Atlantic salmon parr	Not reported	Vancouver Island, British Columbia, Canada	Headkidney	Coding RNAs	Microarrays	Rise et al., (10)	Downregulation of at least 10 genes involved in response to oxidative stress
								Upregulation of complement component
								Altered iron ion homeostasis
								Inflammatory and acute phase responses
								Downregulation of T cell receptor
								Upregulation of C-type lectin and matrix metallo- proteinase
I.P challenge, SW 27.6 ppt, 10,1°C	Atlantic salmon postsmolt	237 g; <2% CV	P. salmonis (PS14LT8) 1 x 10e4 PFU/ml	Liver, headkidney	Coding RNAs	Microarrays	Tacchi et al., (13)	Upregulation of IFN response
								Downregulation of chemokines, chemokine receptors, and an inhibitor of NF-κB
								Downregulation TCR-α, TCR-β, T-cell activation Rho GTPase-activating protein, and CD80
								Upregulation of stress-associated genes
								Downregulation of genes involved in apoptosis
								Upregulation of genes involved in both protein synthesis and protein degradation
								Upregulation of genes involved in energy metabolism
								Downregulation of genes involved in gluconeogenesis
								Downregulation of genes involved in cell signaling mediated by G proteins
I.P challenge; water temperature and salinity not reported	Atlantic salmon	42 g; SD 11 g	P. salmonis LF-89; 0.2 x 10e4.8 TCID 50%/ml	Headkidney and skeletal muscle	Coding RNAs	RNA-seq	Dettleff et al., (105)	Resistant hosts triggered up-regulation of LysC, which may explain a decrease in the bacterial load in head kidney
I.P challenge, 33.92 +- 0.04 ppt; 14.1 +- 0.1°C	Atlantic salmon postsmolt	276.9 +- 78,3 g	P. salmonis LF-89; 1 x 10e4 PFU/ml	Headkidney	Coding RNAs	Microarrays	Pulgar et al., (97)	Upregulation of lysozyme C II
								Upregulation of component of the major histocompatibility complex (MHC) class I
								Upregulation of components linked to the organization and regulation of the actin cytoskeleton, such as cytoplasmic actin, thymosin and tropomyosin
								Downregulation of genes involved in protein synthesis, transport of oxygen and selenium, and homeostasis of metals
								Downregulation of genes involved in intracellular non-hemic iron binding and in hemic binding suggest changes in iron metabolism
I.P challenge; type of water and water temperature no informated	Atlantic salmon	158.3 +- 35,4 g	P. salmonis LF-89; 1 x 10e4 PFU/ml	Headkidney, spleen, brain	Coding RNA; Long non-coding RNA	RNA-seq	Valenzuela-Miranda and Gallardo-Escárate (101)	Clathrin-mediated endocytosis and iron homeostasis
								Endocytic receptors were mainly downregulated
								Strong correlation between the modulations of long non-coding RNAs and genes associated with endocytosis and iron homeostasis
I.P challenge; type of water and water temperature no informated	Atlantic salmon	158.3 +- 35,4 g	P. salmonis LF-89; 1 x 10e4 PFU/ml	Headkidney, spleen	Small non-coding RNA	RNA-seq	Valenzuela-Miranda et al., (100)	Upregulation of genes involved in the immune response, such as cortisol metabolism, chemokine-mediated signaling pathway and neutrophil chemotaxis genes
								miRNA expression in co-modulation with transcription activity of target genes is related to putative roles of non-coding RNAs in the immune response
I.P challenge; type of water and water temperature no informated	Atlantic salmon	154.7 +- 13,5 g	P. salmonis EM-90; 1 x 10e4 PFU/ml	Headkidney, spleen	Coding RNAs	Dual RNA-seq	Valenzuela-Miranda et al., (30)	Both bacteria and host displayed a large number of genes associated with metabolism and particularly related with the amino acid metabolism
								P. salmonis lack of the biosynthetic pathway for several amino acids such as valine, leucine, and isoleucine
								This condition is phenotypically reversed when the amino acids are supplemented in the bacterial growth medium
								There would be a metabolic dependence of P. salmonis on salmon amino acids
Cohabitation challenge, SW 15 PPT, 12°C; cohabitants fish	Atlantic salmon postsmolt	118.4 g	P. salmonis LF-89; 1 x 10e5.6 PFU/ml	Headkidney	Coding RNAs	RT-QPCR	Rozas-Serri et al., (11)	Induction of the inflammatory and IFN-mediated response, modulation of Th1 polarization and reduced antigen processing and presentation
								Modulation of the evasion of the immune response mediated by CD8+ T cells and promotion of the CD4+ T-cell response during the late stage of infection
			P. salmonis EM-90; 1 x 10e5.6 PFU/ml					This response was significantly exacerbated in fish infected by EM-90 isolate, a finding that is probably associated with the higher pathogenicity of EM-90
								P. salmonis is able to manipulate the kinetics of cytokine production to promotes its intracellular survival and replication
Cohabitation challenge, SW 15 PPT, 12°C; cohabitants & shedders fish	Atlantic salmon postsmolt	118.4 g	P. salmonis LF-89; 1 x 10e5.6 PFU/ml	Headkidney	Coding RNAs	RNA-seq	Rozas-Serri et al., (12)	Upregulation of DC-SIGN and TLR5 signaling, which converged at the NF-kB level to modulate the proinflammatory cytokine response
								P. salmonis induced an IFN-inducible response (IRF-1 and GBP-1) but inhibited the humoral and cell-mediated immune responses
								P. salmonis induced significant cytoskeletal reorganization, but decreased lysosomal protease activity and caused the degradation of proteins
								Delayed protein transport, antigen processing, vesicle trafficking and autophagy
			P. salmonis EM-90; 1 x 10e5.6 PFU/ml					Both P. salmonis isolates promoted cell survival and proliferation and inhibited apoptosis
								Both P. salmonis isolates used similar pathways to modulate the immune response in shedders fish at 5 dpi, but the profiles in cohabitants fish were different at 35 dpi
								Regardless of the isolate of P. salmonis, both maintained the viability of host cells and increase intracellular replication and persistence at the infection site
Cohabitation challenge, SW 15 PPT, 12°C; cohabitants, shedders and vaccinated fish	Atlantic salmon postsmolt	118.4 g	P. salmonis LF-89; 1 x 10e5.6 PFU/ml	Headkidney	Coding RNAs	RT-QPCR	Rozas-Serri et al., (30)	Fish infected with LF-89 isolate showed an anti-inflammatory response, but this finding was not observed in the EM-90-infected fish and vaccinated fish
								Fish infected with both P. salmonis isolates showed mhc1-mhc2, cd4-cd8b and igm overexpression
			P. salmonis EM-90; 1 x 10e5.6 PFU/ml					P. salmonis induces IL-10 overexpression and reduces IL-12 expression which could be a strategy to promote intracellular survival and replication
								Vaccinated-fish exhibited mhc1, mhc2 and cd4 overexpression but downregulation of cd8b and igm
			Inactivated whole-cell vaccine 5.7 × 10e5 – 2.5 × 10e6					It is not the same to evaluate the immune response in fish challenged intraperitoneally as by cohabitation
I.P challenge, SW 25 PPT 15°C	Atlantic salmon	40 +- 10 g	P. salmonis LF-89; 1 x 10e4 PFU/ml	Headkidney	Coding RNAs	RT-QPCR	Pontigo et al., (131)	Six mRNA variants of NLRC3 in Atlantic salmon (SsNLRC3) and 2 isoforms were found
								Analysis of six variants involved in the conformation of two different isoforms. Probable function of each isoform in pathogen recognition.
I.P challenge, SW 32 PPT; 15°C	Coho salmon	150 g	P. salmonis LF-89; Doses not reported	Headkidney	DNA methylation	DNA sequencing	Leiva et al., (89)	Genome-wide methylation results disclose significant methylation alterations in coho salmon infected with P. salmonis
								Epigenetic changes observed in the coho salmon genome could be possibly impelled by the bacterial pathogen
I.P challenge, SW 32 PPT; 15°C	Coho salmon	150 g	P. salmonis LF-89; Doses not reported	Plasma-Extracellular vesicles	Small non-coding RNA	RNA-seq	Leiva et al., (132)	Extracellular vesicles-miRNAs target genes showed that they were grouped mainly in cellular, stress, inflammation and immune responses
								P. salmonis could benefit from unbalanced modulation response of coho salmon EV-miRNAs to promote a hyper-inflammatory and compromised immune response
I.P challenge, FW, 15.2°C	Atlantic salmon parr	64.2 +- 10.4 g	P. salmonis EM-90; 10e0.83 TCID50/mL	Headkidney	Coding RNAs	Microarrays RT-QPCR	Xue et al., (99)	Multivariate analyses of infected fish at 21 days-post infection revealed two phenotypes (lower and higher P. salmonis load)
								19 transcripts showed a significant positive correlation with the P. salmonis load: iron metabolism (hampa, frrs1a), inflammatory response (il8a, saa5),
								antibacterial response (campb, c3a) and leukocyte function (ifng, bcl10a).
								6 transcripts showed a significantly negative correlation with the P. salmonis load: oxidative stress response (esn1a, selenopb).

wpc, weeks post-challenge (for cohabitation); wpi, weeks post-infection; dpi, days post-infection; I.p., intraperitoneal; I.m., intramuscular; FW, freshwater; SW, seawater.

In salmonids, the adaptive immune system consists of CMI, whose mechanism of action is to “kill” and eliminate pathogen-infected cells, and humoral immunity, which relies on antibodies to neutralize pathogens in fluids and tissues. As *P. salmonis* is a bacterium that replicates within host cells, it is inaccessible to neutralizing antibodies in the extracellular matrix, so cell-mediated immunity plays a key role. All T cells possess a T cell receptor (TCR) by which they recognize peptides presented by MHC, along with CD3 and costimulatory (CD28) and coinhibitory (CTLA-4) surface molecules (133). T cell-associated genes and their encoded proteins with T cell activity in fish have been well documented (134). The presence of cytotoxic T cells (CTLs) and Th cells in fish have been identified as CD8+ and CD4+ T cells, respectively (135, 136). In the case of CMI against *P. salmonis*, CD8 T cells should recognize infected cells by binding to MHC-I molecules expressing peptides processed from intracellular pathogens. MHC-I ligands bind to their respective TCRs on the surface of CD8+ T cells (137). In addition, CD28 costimulatory or CTLA-4 negative regulatory markers must mediate the interaction between CD8+ cells and MHC molecules (138). Upon binding to MHC-I molecules, naïve CD8 cells are activated into effector CTLs that secrete cytotoxic granules containing perforins and granzymes. Perforins form spores in target cell membranes, enabling granzymes, which are serine protease enzymes, to enter the target cells and cleave to host proteins to induce apoptosis. To execute their effector functions, CD8+ T cells are helped by CD4+ T cells.

The differentiation of naïve CD4+ T cells into Th1 cells is mediated by IL-12 and IFN-γ (139), which play crucial roles in the paracrine and autocrine modulation of APCs in the activation of CD8+ T cells against intracellular pathogens. Therefore, a better understanding of cytokine signaling that promotes CD4+ to CD8+ T-cell differentiation, as well as co-stimulatory signals between APCs and T-cells, is needed to understand the immunomodulators capable of activating a protective CMI response against *P. salmonis*. Antibody responses are normally observed in fish vaccinated against SRS under field conditions, but these humoral immune responses show no correlation with mortality rates and therefore fail to confer protection throughout the production cycle (1, 26, 27). However, although antibodies could react with *P. salmonis* before entering host cells or during cell-to-cell transmission, the real challenge is to develop protective vaccines based on CMI. Rozas-Serri et al. (11, 12) demonstrated that *P. salmonis* induces an IFN-inducible response (IRF-1, GBP-1, IFN), but CD4 overexpression and CD8b underexpression were observed, suggesting that *P. salmonis* modulates CD8+ T cell-driven evasion of CMI and promotes the CD4+ T cell response during the late phase of infection as a mechanism to escape host defenses (**Figure 2**). This mechanism could explain the high bacterial loads, severe pathological lesions, high cumulative mortality, and low survival in *P. salmonis*-infected fish (8). In general, the mechanisms used by intracellular bacteria to trigger CD8+ cell activation have not been fully elucidated in fish, as demonstrated in viral infections (140).

Viruses with economic importance in salmon farming, e.g., ISAV, IPNV, and SAV, are relatively well controlled by vaccines, including conventional vaccines such as virins; these viruses are integrated into a more global strategy that considers the genetic management of resistance, control of production conditions, epidemiological surveillance, and specific regulations for control. ISAV activates a rapid and long-lasting induction of MHC-I pathway genes in Atlantic salmon kidney cells, an effect mediated by virally induced type I IFN (141). These observations suggest that salmon type I IFN has important immunomodulatory functions in activating the MHC-I machinery in response to ISAV infection and that, unlike influenza and many other viruses, ISAV does not seem to interfere with MHC and IFN expression. This could support the thesis that salmon can mount an efficient CMI response, since the viruses are phagocytized, incorporated into antigen-presenting cells, and processed in the cytoplasm of vacuoles and their immunogenic structures are submitted by MHC-I to T cells to finally differentiate into CD8+ T cells. So, what happens to *P. salmonis* if it also replicates within vacuoles in the cell’s cytoplasm? When the bacterium is alive and infects fish, it is plausible that the bacterium’s virulence factors support its strategy to escape antigen processing and/or that MHC-I does not present the antigens efficiently, and the bacterium finally succeeds in evading the fish’s response mechanisms. The next question would then be whether this is the reason why fish are also unable to activate a response against a bacterin-like vaccine or even a live attenuated vaccine. Hence, elucidating which *P. salmonis* proteins are involved in altering the processing and/or presentation of its antigens in host cells and how they act could provide information to confirm the hypothesis that the bacterium is able to alter this cellular mechanism to evade CMI.

This could presumably be because of the selective pressure exerted by the immune system, many viruses have evolved proteins that interfere with antigen presentation by MHC-I molecules (142). Viral proteins have been characterized to exploit bottlenecks in the MHC-I pathway, such as peptide translocation by transporters associated with antigen processing (143). Alternatively, viral proteins can cause the degradation or mislocalization of MHC-I molecules. This is often achieved by the subversion of the host cell’s own protein degradation and trafficking pathways. Antigen processing and presentation by MHC molecules is a cornerstone in vertebrate immunity. Six different MHC-I lineages have been described in teleosts: U, Z, S, L, P, and H (144, 145). Although structurally similar to classical MHC-I molecules, all belong to the U lineage (Sasa-uba), and many nonclassical/MHC-I-like molecules (L lineage genes *Sasa-lda, Sasa-lia, Sasa-lca, Sasa-lfa, Sasa-lga*, and *Sasa-lha*) have functions other than peptide presentation, ranging from host homeostasis to immune regulation (146). Using two separate *in vivo* challenge models in Atlantic salmon with different kinetics and immune pathologies combined with *in vitro* stimulation using viral and bacterial TLR ligands, Svenning et al. (147) showed that *de novo* synthesis of different L lineage genes is distinctly regulated in response to different types of immune challenges. In this way, while salmonid alpha virus 3 (SAV3) strongly induced the expression of *lia*, *lga* and, to a lesser extent, *lha*, but not *lda*, *lca* or *lfa*, infection by *P. salmonis* resulted predominantly in positive regulation from *lga*. The induction of *lca*, which is predominantly expressed in primary and secondary lymphoid tissues, was marginal except for a transient upregulation in the pancreas following SAV3 challenge.

## Vaccination Against *P. salmonis*: Mission Possible?

Biosecurity, vaccination, and selective breeding for disease resistance are the main tools for controlling infectious diseases in aquaculture. However, host genetics and vaccines provide only partial protection, raising concerns about their effectiveness in the field. Pathogens can be divided into extracellular, facultative intracellular, and obligate intracellular pathogens. Intracellular pathogens pose special challenges to the immune system because regardless of whether they are exposed extracellularly, they cannot be contacted directly by immune cells or by humoral factors such as antibodies. The aim of vaccination is to train the adaptive immune system of fish by exposure to pathogenic antigens so that upon subsequent exposure, the immune system mounts a rapid and long-term protective response against the same pathogen. The success of vaccination against bacterial diseases in fish is mainly attributed to vaccines targeting extracellular pathogens, as they activate antibody-mediated humoral adaptive immunity (148). However, circulating antibodies do not protect against *P. salmonis* infection or disease development, because *P. salmonis* is a facultative intracellular bacterium. Stimulation of the antigen presentation and processing process of *P. salmonis* is therefore the initial target of vaccines, which then aim to modulate immunological memory; but it is precisely these processes that constitute one of the current limitations in fish. Although IFN-γ has been reported to promote the expression of APC surface markers that are key in co-stimulating the signal for T-cell activation and Th1 polarization, it would also promote polarization towards Treg (94), indicating an immune tolerance profile that needs further investigation because it could be the cause of inhibition of the inflammatory response to promote an anti-inflammatory response that determines persistent infection and chronic disease course in fish. It is not known whether an increased inflammatory response driven by highly destructive proinflammatory macrophages, although they are an important part of Th1 polarization, could drive a CMI capable of controlling *P. salmonis* evasion mechanisms. On contrary, exacerbated activation of the IFN-γ-mediated inflammatory response (11, 12) appears to increase the susceptibility of infected fish, culminating in progression of the response from an acute to a chronic stage and resulting in increased intracellular replication of *P. salmonis*, high bacterial loads, serious pathological lesions and low survival (8). In addition, Iliev et al. (149) showed that the potent immunostimulatory properties of a TLR ligand would not necessarily translate into enhanced APC functions and highlight the complexity of the activation of fish immune cells by TLR ligands.

That said, there is a need to modulate and activate the CMI response, but available vaccines do not trigger effective antigen presentation through MHC-I, and there is a lack of adequate activation and expansion of T cells, especially CD8+ cytotoxic T cells (29, 38). The efficacy of vaccines reported from experimental challenges has always been acceptable (1, 25), but current vaccination strategies under field conditions, whether based on replicating or nonreplicating vaccines, have shown only a transient activation of the humoral immune response and specifically of the CMI response, which are not strong or long-lasting enough to achieve effective control of SRS (1, 26, 27, 29, 38). Based on current understanding of the limitations of vaccines for SRS control and the intracellular nature of *P. salmonis*, other vaccines may be advantageous. A new immunoinformatics-based strategy to design vaccines using epitopes of antigenic proteins from bacteria and viruses that directly promote T- and B-cell activation and differentiation has been used in humans. Although it is not yet a popular strategy in fish, the design and development of multi-epitope vaccines against *Flavobacterium columnare* (150), *E. tarda* y *F. columnare* (151), *Streptococcus agalactiae* (152), and *E. ictaluri* (153) have been reported. This in silico strategy was used by a consortium of Chilean entities to design a multi-epitope chimeric vaccine targeting different epitopes of *P. salmonis*.

The variable and relative protection of current vaccines against SRS under field conditions could be a consequence of different environmental variables (1), the choice of the vaccination strategy (26, 27, 137), coinfection with other pathogens such as sea lice (154) or the variation in genetic resistance to SRS among families (155). In this way, different factors related to fish production can lead to increased susceptibility, coinfection, and mortality. Everson et al. (156) showed the importance of environmental factors, host genetics, and vaccination in relation to infection and mortality related to infectious diseases. However, the main reason would be related to insufficient and/or transient activation of the CMI response induced by conventional vaccines (1, 10–13, 25, 29, 38, 97, 105). Thus, translating recent knowledge about fish-*P. salmonis* interactions, specifically about the immune response and its pathogenesis, into better vaccines or better vaccination strategies is not an easy task and remains one of the main challenges in fish vaccinology.

The kinetics of the antibody response are transient after freshwater vaccination, and antibody levels begin to decline approximately 1800 days postvaccination (22). However, the mechanisms that could explain this fact are the natural challenge and exposure of fish to *P. salmonis* and, consequently, the consumption of antibodies, as has been observed in salmon vaccinated with IPN (140). These results supported the idea of complementing the classical parenteral vaccination strategy with the application of a vaccine booster administered orally before the fall of the antibody titer (22). However, the field results obtained from several generations of farmed salmon were not as expected, and this strategy did not contribute significantly to disease control (1, 25, 29, 38). The intraperitoneal route is usually chosen for the administration of vaccines in salmon, but there is little knowledge about its immune response. Recently, a significant increase in leukocytes, total IgM antibody-secreting cells (ASCs) and *P. salmonis*-specific ASCs in the peritoneal cavity at 3 and 6 weeks after infection with *P. salmonis* has been described (157). Hence, the authors suggest a putative role for adipose tissue in the peritoneal cavity immune response.

The microbiome plays an important role in the maturation of the vertebrate adaptive immune system and stimulation of the immune response and can directly enhance the host pathogen defense *via* colonization resistance and the production of inhibitory compounds (158). Complementarily, the mucosal immune response based on IgT+ B cells and secreted IgT plays a key role, as IgT is highly induced by pathogens on mucosal surfaces and coats much of the fish microbiota (34, 159, 160). However, there is an important gap regarding how to complement systemic immunity against *P. salmonis* with advances in the knowledge of activating local immunity at the site(s) of *P. salmonis* entry, such as the gills and skin, as well as the gut, which could help limit the success of infection.

Complementarily, selective breeding for improved resistance to infectious diseases is a potentially a more sustainable strategy for the long-term control of disease outbreaks in aquaculture (161), especially when vaccination strategies have not been as effective as expected. Previous studies have demonstrated significant genetic variation for resistance to *P. salmonis* in Atlantic salmon (h^2^ = 0.11 to 0.41), rainbow trout (h^2^ = 0.45 to 0.62) and Coho salmon (h^2^ = 0.16) using disease challenge data (162–167). There is an important genetic component of SRS resistance in Atlantic salmon supported by a polygenic architecture. When comparing the response between fish with high and low resistance to *P. salmonis*, changes in the expression of genes related to the cytoskeleton, apoptosis and cell survival, bacterial invasion/intracellular trafficking and the inflammasome are observed, which correlate with genetic resistance (168). Thus, the possible mechanisms leading to genetic resistance are likely heterogeneous and vary among different families and individuals.

One SNP related to B cell development was identified as a potential functional candidate associated with resistance to *P. salmonis* defined as days to death (162). B cells are critically important in the humoral immune response, but they are also dedicated phagocytes, so the latter innate function could be important in the immune response against *P. salmonis* in Coho salmon. Early growth is positively correlated genetically with resistance to *P. salmonis*, measured by the day to death and by the harvest weight in Coho salmon, so selective breeding for early growth could indirectly improve the harvest weight and resistance to *P. salmonis* in the population (169).

According to Figueroa et al. (155), the between-familiar variation is a strong intrinsic factor that determines the variable protection of vaccines for SRS. In some full-sibling families, the added protection by vaccination increased the survival time compared to their unvaccinated siblings; in other families, there was no added protection by vaccination. However, further studies are needed to assess whether variations in the host immune response to vaccination for SRS control could explain the differences in resistance observed among vaccinated fish and what biological mechanisms could explain it, because vaccination and selective breeding have always been considered supplementary strategies in disease control in aquaculture. In addition, although cohabitation challenge models provide little information on whether fish with higher genetic resistance and/or vaccinated fish are less likely to transmit infection when infected, they are more suitable models than intraperitoneal challenge models for evaluating disease resistance and/or vaccine efficacy.

Mortality is the main phenotype for disease resistance in fish, but there is emerging evidence that directly targeting survival by breeding or vaccination without considering the epidemiological effects does not reduce disease transmission or the disease prevalence at the population level (170–172). Three key epidemiological host traits affect the infectious disease prevalence and population mortality rates are susceptibility, infectivity, and endurance (170). Chase-Topping et al. (173) reported that both vaccination and genetic selection reduce but do not prevent ISA transmission. The authors suggest that it would be very beneficial to assess the combined effects of genetic selection and vaccination on ISA transmission and to evaluate the mucus viral load as a potential *in vivo* indicator for individual’s infectivity. These results should be applied to SRS transmission experiments to understand the effects of vaccination strategies and genetic resistance on SRS control under field conditions. Similarly, since host genetics and vaccines provide only partial protection and raise questions about their efficacy in the field, the control plan for SRS must consider the epidemiological edge, as little is known about the impact of genetic management and/or vaccines on the transmission and prevalence of SRS. Based on this scenario, it could be hypothesized that relative control of SRS is based on regulatory and production changes designed and implemented by industry due to an evolving public–private roadmap, which includes vaccination strategies but also a low relative contribution.

## Concluding Remarks and Future Avenues

Infectious diseases caused by intracellular bacteria in different farmed fish species worldwide represent a great challenge, especially for their control by vaccination and genetic resistance to disease. One of these diseases is SRS, which has been described in several salmon-producing countries, but which especially affects Chilean salmon farming. In practice, given the industry’s collective imperative need to advance in the control of SRS in order to increase its long-term sustainability and competitiveness, it seems a frivolity to discuss whether *P. salmonis* is a pathogenic or environmental bacterium (174, 175), since, in either case, reality shows us that: (1) *P. salmonis* is a very complex bacterium that we need to more about, especially its interaction with host cells; (2) SRS is the main infectious disease of Chilean salmon farming, generating economic losses conservatively estimated at approximately 700 million dollars per year; and (3) an average of 93.28% of total antibiotics used in the Chilean salmon industry in the last 5 years has been only for the control of SRS. That said, it is undoubtedly the main mission of all members of the salmon industry to continue working together to fill the knowledge gaps that will allow us to optimize the control of SRS; with a specific focus on the interaction of the components of the epidemiological triad: the pathogen, the fish and the aquatic environment (including production management).

Significant knowledge has been generated in recent years on different virulence factors of *P. salmonis* that could explain the difficulties in its control under field conditions: (1) Sec-dependent pathway and Type 4 Dot/Icm secretion system; (2) expression of ClpB and BipA proteins; (3) siderophore production; (4) genes for vibroferrin biosynthesis; (5) pathogenicity islands; (6) membrane vesicles; (7) large cryptic plasmids; (8) Sec dependent pathway; (9) type IV pili and biofilms; and (10) expression of virulence-associated genes during stringent response, among others. However, more specific knowledge is still required regarding the mechanisms of action of the virulence factors of *P. salmonis* that give it a high capacity to evade the host response. New methods in genetics and molecular biology have contributed to the *in vitro* study of the basic biology of *P. salmonis*. Although not all genes of pathogenic microorganisms play a role in their pathogenicity or virulence, nor can they be confirmed only in a cell culture invasion model. Methods such as subcellular fractionation and single-molecule super-resolution microscopy should be applied to tag molecules inside bacteria and host cells to follow their fate during early and late infection, and even to find *P. salmonis* genes or proteins that are activated only when they are inside cells.

Although effective control of infectious diseases in aquaculture requires the integration of several management measures and interventions, there is a consensus that the availability of an effective vaccine(s) should be one of the most important pillars to support control. However, although there is evidence that efforts have been made in the development of vaccines against SRS, unfortunately, the objective has not yet been achieved, and the SRS control plan basically lacks an effective vaccine. Existing evidence shows that the production of CMI-based vaccines would be the most effective approach to reduce the prevalence and severity of SRS outbreaks. Given that this is one of the most virulent bacterial pathogens for salmon, continued research on how *P. salmonis* evades fish adaptive responses is necessary to lead to new insights that will improve our understanding of host-pathogen interactions.

Basically, the strategy for developing effective vaccines against *P. salmonis* must change, starting with understanding the biological pathways that the bacterium uses to infect and replicate in host cells and then defining strategies to block them. The conventional approach historically used for vaccine development against *P. salmonis* has not been the solution to the problem, which is why salmon producers have no other alternatives in the field when faced with SRS outbreaks other than to use the last card they have available, chemotherapy. Consequently, the time has come for new generation vaccines and reverse vaccinology in aquaculture: DNA, mRNA, viral vectors, multiepitope and multiantigenic chimeric vaccines, among others. However, it is necessary to generate deeper and more specific knowledge about the interaction between *P. salmonis* and fish cells and, of course, to generate the appropriate regulatory instances for the formal registration of these new vaccines. The governmental agencies in charge of regulating the vaccine registration system and the control of its application in field conditions must advance as fast as the new knowledge and products from research and innovation are generated. It is not prudent to develop new generation vaccines without a regulatory structure in place to authorize and regulate their commercial use in advance.

From a bacterial-host cell interaction point of view, more is known about *P. salmonis*-induced changes in cell invasion and intracellular survival, but we are only beginning to understand how the immune system detects and responds to these changes. Considering these new data, several important gaps emerge: (1) Does the role of the cytoskeleton in cell-autonomous immunity differ between phagocytes or epithelial cells? Further study is required to determine how cytoskeletal components in cell-autonomous immunity—actin, microtubules, intermediate filaments and septins—work together during *P. salmonis* infection. (2) Immunological speaking, more specific knowledge should be promoted to understand the mechanisms of processing and presentation of *P. salmonis* antigens in APCs, macrophages and DCs, to T-cells. Further knowledge about fish macrophages and DCs biology is needed to better understand how *P. salmonis* modulates their biology to its own advantage. Does *P. salmonis* prevent the presentation of its antigens by MHC-I? Do APCs activate T cells in the quantity and quality necessary to detect and eliminate *P. salmonis*-infected cells? How do APCs activate salmon T cells, and how do we quantitatively define the efficacy of the T cell activation process to be considered a protective response? What is the role of B cells as phagocytes and professional antigen-presenting cells in *P. salmonis* infection? How can the *in vivo* mechanism of action of the main virulence factors of *P. salmonis* be blocked? What is the mucosal immune response against *P. salmonis* and the role of the microbiota?. (3) What is the genetic variability of fish in terms of their immune response against *P. salmonis*? How do genetic resistance management and vaccines complement each other in the control of SRS? Why do these two strategies not reduce the transmission and prevalence of SRS? Which epidemiological variables are we not considering in the control of SRS, or which variables are we not giving enough relevance to? The million-dollar question will remain whether effective control of SRS will be achieved even if vaccines that trigger effective CMI responses are developed. The challenges of inducing CMI responses against *P. salmonis* are considerable, as are the challenges presented by other intracellular pathogens in aquaculture, such as *Francisella*, *Renibacterium* and *Edwarsiella*. The importance of CMI, which consists of the production of natural killer cells, CD8 cytotoxic T cells and CD4+ Th1 cells, is critical for protection against *P. salmonis* through proper activation of mononuclear phagocytes. Strategies to increase resistance to the bacterium could focus on altering its modulation of cellular homeostasis (cytoskeleton, apoptosis, or cell cycle progression) or enhancing immune processes that prevent or slow infection (inflammasome, antigen recognition and presentation, T cell activation and proliferation, increased cytotoxic capacity of CD8+ T cells, oxidative stress), among other pathways. These strategies may include knockout or CRISPR/Cas modulation in cell line models or, ultimately, *in vivo* to interrogate the impact of disruption of identified genes on genetic resistance.

## Author Contributions

The author confirms being the sole contributor of this work and has approved it for publication.

## Funding

The author declare that this study received funding from Pathovet Labs. The funder was not involved in the study design, collection, analysis, interpretation of data, the writing of this article or the decision to submit it for publication.

## Conflict of Interest

Author MR-S was employed by the company Pathovet Labs.

## Publisher’s Note

All claims expressed in this article are solely those of the authors and do not necessarily represent those of their affiliated organizations, or those of the publisher, the editors and the reviewers. Any product that may be evaluated in this article, or claim that may be made by its manufacturer, is not guaranteed or endorsed by the publisher.
